# Increase in tibial internal rotation due to weight-bearing is a key feature to diagnose early-stage knee osteoarthritis: a study with upright computed tomography

**DOI:** 10.1186/s12891-022-05190-3

**Published:** 2022-03-15

**Authors:** Kazuya Kaneda, Kengo Harato, Satoshi Oki, Yoshitake Yamada, Masaya Nakamura, Takeo Nagura, Masahiro Jinzaki

**Affiliations:** 1 Department of Orthopedic Surgery, Keio University School of Medicine, 35 Shinanomachi, Shinjuku, Tokyo, 160-8582 Japan; 2 Department of Diagnostic Radiology, Keio University School of Medicine, Tokyo, Japan

**Keywords:** Upright computed tomography, Early knee osteoarthritis, Weight-bearing, Surface registration

## Abstract

**Background:**

The classification of knee osteoarthritis is an essential clinical issue, particularly in terms of diagnosing early knee osteoarthritis. However, the evaluation of three-dimensional limb alignment on two-dimensional radiographs is limited. This study evaluated the three-dimensional changes induced by weight-bearing in the alignments of lower limbs at various stages of knee osteoarthritis.

**Methods:**

Forty five knees of 25 patients (69.9 ± 8.9 years) with knee OA were examined in the study. CT images of the entire leg were obtained in the supine and standing positions using conventional CT and 320-row detector upright CT, respectively. Next, the differences in the three-dimensional alignment of the entire leg in the supine and standing positions were obtained using 3D-3D surface registration technique, and those were compared for each Kellgren–Lawrence grade.

**Results:**

Greater flexion, adduction, and tibial internal rotation were observed in the standing position, as opposed to the supine position. Kellgren–Lawrence grades 1 and 4 showed significant differences in flexion, adduction, and tibial internal rotation between two postures. Grades 2 and 4 showed significant differences in adduction, while grades 1 and 2, and 1 and 3 showed significant differences in tibial internal rotation between standing and supine positions.

**Conclusions:**

Weight-bearing makes greater the three-dimensional deformities in knees with osteoarthritis. Particularly, greater tibial internal rotation was observed in patients with grades 2 and 3 compared to those with grade 1. The greater tibial internal rotation due to weight-bearing is a key pathologic feature to detect early osteoarthritic change in knees undergoing osteoarthritis.

## Introduction

The classification of knee osteoarthritis (OA) is an essential clinical issue, particularly in terms of the diagnosis of early knee OA, as the primary goals of treatment of knee OA is to prevent its progression and to avoid irreversible degenerative change occurring in the joint [1]. To apply effective intervention to early knee OA, precise diagnosis is a first step. However, there is a limitation to use two-dimensional (2D) radiographs to evaluate the degree of tibiofemoral joint deformity in early-stage knee OA. Traditionally, the Kellgren–Lawrence (K–L) classification, which was developed in 1957, is considered the gold standard for the clinical evaluation of knee OA and is used for radiological grading [2]. This classification is based on osteophyte formation and joint space narrowing, and subtle changes in these features are difficult to determine on 2D images. Several studies have suggested limitations of K-L classification in diagnosis of early knee OA [3–6] To overcome this problem, Oka et al. developed an automatic system for diagnosing knee OA (knee osteoarthritis computer-aided diagnosis; KOACAD) [7] and reported normal and threshold values of various knee OA parameters [8]. Although KOACAD enables the automatic classification of knee OA, the joint space widths between K–L1 and K–L2 differ by <0.4 mm, [8] which is too small to enable differentiation on clinical radiographic images. Thus, the ability to diagnose early degenerative changes in the tibiofemoral joint based on 2D radiography remains limited.

In recent years, weight-bearing computed tomography (CT) has been investigated as a potentially more reliable method for diagnosing knee OA. For example, Segal et al. reported the superior test-retest reliability of cone-beam CT for the three-dimensional (3D) measurement of joint space width in the weight-bearing OA knee, with intraclass correlation coefficients of 0.95–0.97 and 0.90–0.97 for the lateral and medial compartment, respectively [9, 10]. Hirschmann et al. also used cone-beam CT to determine the effect of weight-bearing using 3D images of the knee joint [11]. However, these studies conducted cone-beam CT using effective fields of view of 220 mm × 220 mm or 200 mm × 350 mm, respectively, which were not sufficiently wide to scan the entire leg or evaluate the lower limb alignment while standing. Accordingly, the use of cone-beam CT to conduct a 3D analysis of the tibiofemoral deformity has been limited. Recently, Fujii et al reported that 3D lower limb alignment under weight-bearing condition [12]. Their results clearly demonstrated 3D deformity of knees undergoing OA, and suggest that it is important to evaluate 3D whole leg alignment under weight-bearing when diagnosing OA.

Our institution has installed a 320-detector upright CT capable of scanning the entire body in a standing position [13–16]. In order to know the condition of the knee joint, it is necessary to know the alignment of the whole leg, especially under weight-bearing. As mentioned above, radiographs and partial cone-beam CT have been used to evaluate joint space narrowing under weight-bearing, but it has been difficult to evaluate whole leg alignment under weight-bearing in 3D using those imaging modalities. In this study, we aimed to use the upright CT and conventional CT to evaluate changes in the 3D alignment of the entire leg in response to weight-bearing in patients with medial knee OA. We hypothesised that weight-bearing causes 3D alignment changes of the knee even in early-stage knee OA.

## Methods

### Subjects

A power analysis was performed to calculate the minimum sample size for the study, as our study design involves two CT examinations for each subject. We used population values and the sigma of our previous study [14] and calculated the sample size with an alpha probability of 0.05, effect size of 0.5, and power of 0.8. The analysis revealed that a minimum sample size of 48 was required for the study. According to the analysis, we recruited 26 patients to study 52 knee joints. We recruited patients with knee OA being treated at our hospital in writing and included those who offered to participate in the study. However, 7 knees in 6 patients diagnosed with inflammatory arthritis on MRI or having valgus knee OA were excluded as this study focused on medial knee OA. Finally, we included 45 knee joints in 25 patients with knee OA (21 women, 4 men). Knee OA was defined as knee joint pain or stiffness during the past 3 months in the absence of any trauma including medial and lateral knee ligament injuries or systematic disease (e.g. rheumatoid arthritis) that could cause knee joint pain as well as a radiological classification of a K–L grade ≥1 based on an anterior-posterior radiograph obtained under the supine position [2]. Three orthopaedic surgeons who have more than 20 years of experience in treatment of knee OA diagnosed and classified the patients. All methods were performed in accordance with the Japanese Ethical Guidelines for Medical and Biological Research Involving Human Subjects. Each participant provided written informed consent, and the study protocol was approved by our ethical committee (Institutional Review Board ID# 20150293).

### Image acquisition

**Fig. 1:**
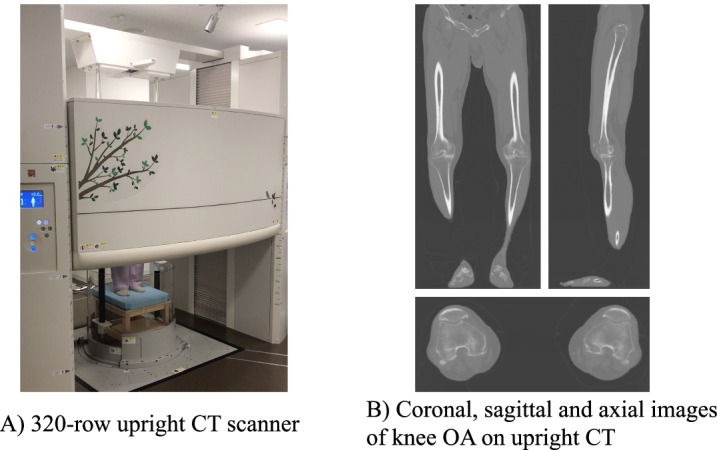
Upright computed tomography (CT) scanner and the acquired images. **A** A 320-row upright CT scanner (prototype TSX-401R; Canon Medical Systems, Otawara, Japan) was used to acquire images from the hip joint to the end of the foot in the standing position. The approximate acquisition time was 10–20 s. **B** Acquired images of knee osteoarthritis (OA) with an upright CT scanner. The image qualities of the upright CT scanner range from good to excellent [11]

CT images were acquired from the pelvis to the end of the foot using two CT scanners under the following conditions. CT examinations in the supine position were performed using a 320-row CT scanner (Aquilion ONE, Canon Medical Systems, Otawara, Japan), while examinations in the standing position were performed using an upright CT scanner with a 320-row detector (prototype TSX-401R, Canon Medical Systems; Fig. 1A). The patients stood or lay in a relaxed position and placed both feet at shoulder width. To avoid intentional rotation of the hip joints, the patients were only instructed to extend the knees as much as possible, with no other restrictions. The upright CT examinations were performed using the following parameters: peak tube voltage, 100 kV; tube current, 10–800 mA (noise index = 8 at a slice thickness = 5 mm); rotation speed, 0.5 second ; and slice thickness, 0.5 mm (Fig. 1B). The approximate acquisition time was 10–20 seconds.

### Surface registration

**Fig. 2:**
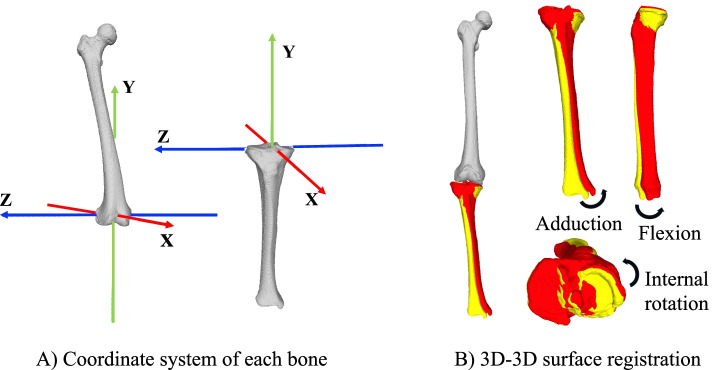
Coordinate systems of each bone. **A** Coordinate system of the tibia was defined as reported by Sato et al. and the International Society of Biomechanics. The rotations around X, Y, and Z were defined as the varus/valgus, external/internal rotation, and flexion/extension, respectively. **B** After three-dimensional (3D)–3D surface registration of the femur, the relative movement of the tibia due to weight-bearing was evaluated in the sagittal, coronal, and axial planes

The 3D surface data corresponding to the femur and tibia were extracted from the CT DICOM files using 3D visualisation software (AVIZO 6.4; Thermo Fisher Scientific, Tokyo, Japan). For each participant, we matched the femoral surfaces in both positions using the iterative closest point (ICP) algorithm from the Visualization Toolkit 8.1.0 (Kitware Inc., Clifton Park, NY, USA) for the 3D surface registration technique (Fig. 2B). Besl and McKay proposed a 3D–3D surface registration technique using this ICP algorithm in 1992 [17]. In a previous in vivo 3D lumber spine study using this technique, the mean absolute translation error was observed to be <0.1 mm in the x-direction and z-direction, and the mean absolute rotation error was <0.2° around the X-axis and Z-axis [18]. We set the number of registration times by ICP algorithm to be 1000 times and performed 3D-3D surface registration.

### Coordinate System

We used a modified femoral and tibial coordinate system based on the method defined by Sato et al. [19], Enomoto et al. [20] and the International Society of Biomechanics [21] (Fig. 2A). For the femoral coordinate system, the Z-axis was defined as a rightward-pointing line connecting the centres of the posteromedial and posterolateral condyles. The temporary (T)-axis was defined as a line connecting the centre of the Z-axis and the centre of the femoral head. The X-axis was defined as the line perpendicular to both the Z-axis and T-axis and pointing anteriorly. The Y-axis was defined as an upward-pointing line perpendicular to both the Z-axis and X-axis.

For the tibial coordinate system, the X-axis was defined as an anterior-pointing line connecting the proximal end of the tibial tuberosity medial margin and the centre of posterior intercondylar notch at the level of lateral joint space (Akagi’s line [22]). The T-axis was defined as an upward-pointing line connecting the centre of the Akagi’s line and the centre of the tibial plafond. The Z-axis was defined as a laterally pointing line perpendicular to both the X-axis and T-axis. The Y-axis was defined as an upward-pointing line perpendicular to both the X-axis and Z-axis.

### Analysis of Joint Movement

Next, we analysed differences in knee rotation between the supine and standing positions. We used the Euler/Cardan angles representing three sequential rotations about the anatomical axis of the proximal bone to describe the bone-to-bone rotations of the tibia relative to the femur around each axis. Specifically, we used the Euler rotation sequence Z-X-Y, wherein the rotations around X, Y, and Z were defined as varus/valgus, external/internal rotation, and flexion/extension, respectively.

In addition, to evaluate the difference in the original lower limb position in the supine and standing positions during the CT examinations, the tibia external angles with respect to the pelvis were calculated. Specifically, the angle between the perpendicular of the line connecting both anterior superior iliac spins of the pelvis and the anterior–posterior axis (X-axis) of the tibia was measured.

### Statistical Analysis

Participants were divided into four grades based on the K–L classification: K–L grade 1, K–L 2, K–L 3, and K–L 4 [2]. Differences in the knee angles in each plane were compared among these four grades. To examine the validity of the rotation of the foot position, we examined the correlation between the tibia external rotation angle with respect to the pelvis and the tibia external rotation angle with respect to the femur, and we also compared the four grades. To compare the four grades, we used ANOVA at a significance level of *p* < 0.05. SPSS 24.0 (IBM, Armonk, NY, USA) was used for statistical analysis.

## Results

**Fig. 3:**
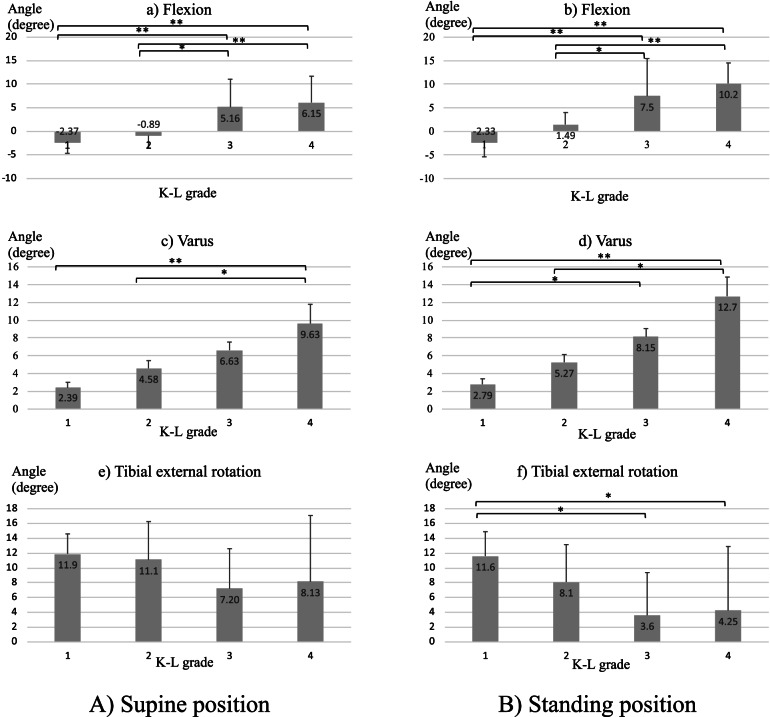
Comparison of the 3D rotation angle among OA grades in both the supine and standing positions. An asterisk (*) indicates a significant difference (*p*<0.05), a double asterisk (**) indicates a significant difference (*p*<0.01). **A** In the supine position, the knee joint flexed and adducted in the high K-L grade group compared to the low K-L grade group, but there was no significant difference in rotation. **B** In the standing position, the OA knee joint flexed and adducted in the high K-L grade group, and the tibia was originally externally rotated with respect to the femur but gradually internally rotated in the high K-L grade group

**Fig. 4:**
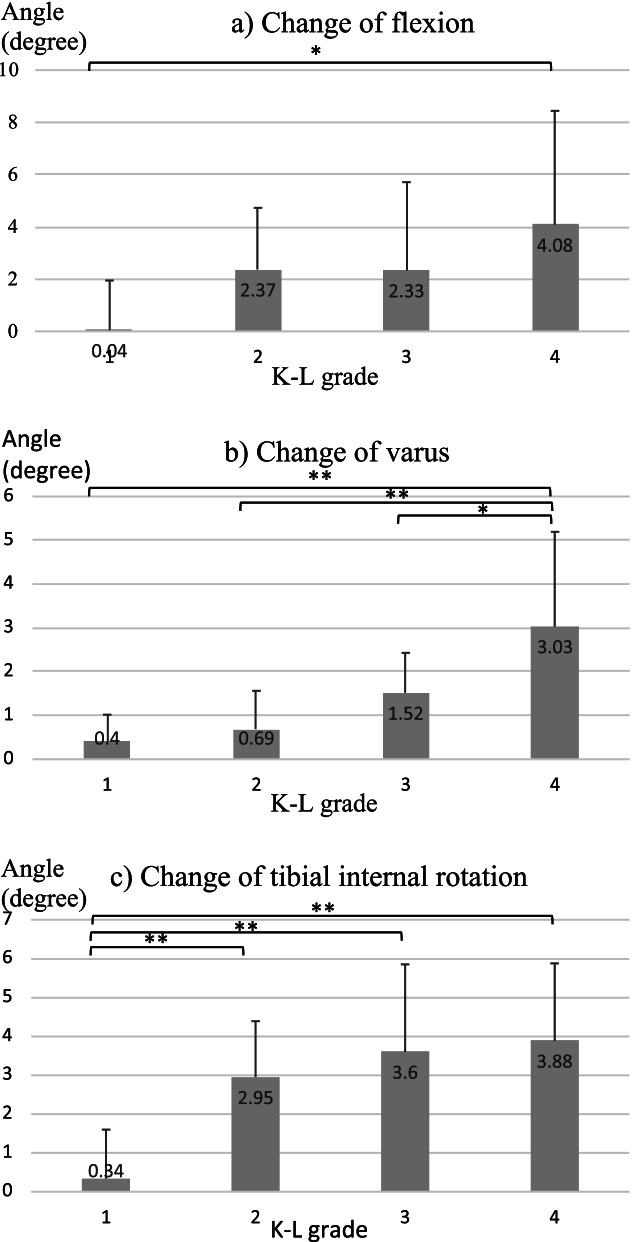
Differences in sagittal, coronal and axial angles of the knee between supine and standing. An asterisk (*) indicates a significant difference (*p*<0.05), a double asterisk (**) indicates a significant difference (*p*<0.01). **a** Flexion angle by K-K grade. **b** Varus angle by K-L grade. **c** Tibial internal rotation angle by K-L grade. The change in the flexion angle differed significantly between K–L grades 1 and 4 (*p* < 0.05). Changes in the adduction angle differed significantly between K–L grades 1 and 4 and between grades 2 and 4 (*p* < 0.01). Changes in the internal rotation angle differed significantly between K–L grades 1 and 2, between grades 1 and 3, and between grades 1 and 4 (*p* <0.01)

Based on the radiographs, 11, 11, 11, and 12 knees were categorised as K–L grades 1, 2, 3, and 4, respectively. The mean (± standard deviation) age, body weight, and body mass index of the participants were 69.9 ± 8.9 (range, 53–86) years, 58.5 ± 12.8 (range, 36.0–80.0) kg, and 24.3 ± 5.1 (range, 16.9–35.3) kg/m^2^, respectively. For the comparison of all coordinate axis rotations, ANOVA results showed *p* ≥0.05 for Levene’s test, so equal variances were used and Tukey’s test was performed as a post-hoc test. In the supine position, the knee joint flexed and adducted in the high K-L grade group compared to the low K-L grade group, but there was no significant difference in rotation. In the standing position, the OA knee joint flexed and adducted in the high K-L grade group, and the tibia was originally externally rotated with respect to the femur but gradually internally rotated in the high K-L grade group (Fig. 3). The flexion angles between the two positions in K–L grades 1, 2, 3, and 4 changed by 0.04 ± 1.91°, 2.37 ± 2.36°, 2.33 ± 3.38°, and 4.08 ± 4.36°, respectively (Fig. 4a). The difference between K–L grades 1 and 4 was significant (*p* = 0.020). The varus angles between the two positions in K–L grades 1, 2, 3, and 4 changed by 0.40 ± 0.62°, 0.69 ± 0.87°, 1.52 ± 0.91°, and 3.03 ± 2.16°, respectively (Fig. 4b). The differences between K–L grades 1 and 4 (*p* <0.001), between grades 2 and 4 (*p* <0.001) and between K–L grades 3 and 4 (*p* =0.042) were significant. The tibial internal rotation angles between the two positions in grades K–L 1, 2, 3, and 4 changed by 0.34 ± 1.26°, 2.95 ± 1.44°, 3.60 ± 2.25°, and 3.88 ± 2.00°, respectively (Fig. 4c). The differences between K–L grades 1 and 2 (*p* = 0.008), between grades 1 and 3 (*p* =<0.001), and between grades 1 and 4 (*p* < 0.001) were significant. No other differences were found in the 3D rotation angle among the 4 OA grades.

Table 1 presents difference in lower leg positions under two CT examinations, that were the tibia external rotation angles with respect to the femur and pelvis. Pearson’s correlation coefficient between the tibia external rotation angle with respect to the pelvis and the tibia external rotation angle with respect to the femur were both 0.069, and there were no significant correlations (*p* = 0.52). Also, there was no significant difference in tibia external rotation angle with respect to the pelvis between each grade between the supine and standing positions during the CT examinations.

**Table 1:** The tibia external rotation angle with respect to the pelvis

KL	Supine	Standing
1	13.9 ± 9.30	7.48 ± 11.5
2	17.4 ± 18.6	3.74 ± 10.7
3	22.8 ± 14.5	13.1 ± 7.78
4	20.9 ± 13.0	10.4 ± 9.43

Data are expressed as mean ± SD

## Discussion

Our findings from this study supported our hypothesis that weight-bearing causes 3D alignment changes of the OA knees, and greater 3D deformities were found in knees with end-stage OA. An important finding of our investigation was the determination of a significantly greater tibial internal rotation with respect to the femur under weight-bearing conditions in knees with K–L grade 2 OA, compared to grade 1 OA. In K-L grade 2, there is subtle joint space narrowing with minimal osteophyte formation on the anterior-posterior radiography [2, 7, 8]. While weight-bearing CT clearly detects early change occurring in the knee joint which is increased internal rotation with weight-bearing.

Consistent with our findings, Hirschmann et al. used cone-beam CT to demonstrate that the tibial internal rotation increased in the knee under weight-bearing conditions [11]. However, that study was significantly limited by the following factors: the subjects without knee OA were involved (17 of 26 were knee OA) ; a relation between the OA grade and tibial internal rotation was not found; the axial CT images were subjected to 2D measurements, which should include measurement errors to account for possible differences in the levels of axial CT images; and an unnatural subject standing posture (i.e. standing on the foot of the examined leg with the other knee bent and resting outside the gantry) that placed the knee in a non-physiological position. Matsui et al. used conventional CT to evaluate rotational deformity in patients with knee OA in supine position and observed an increase in the tibial external rotation as the OA severity increased [23]. However, a direct comparison of our results with their results is difficult, as the OA knees in their study were more severely deformed (femorotibial angles > 190° or 200°) than those in our study, and the two studies used different coordinate systems to measure tibiofemoral rotation. Recently, Fujii et al. compared 3D lower limb alignment in OA knees between weight-bearing and non-weight-bearing conditions [12]. They used 2D–3D image-matching with biplanar computed radiography (CR) and 3D bone models of the complete lower extremity rebuilt using computed tomography-based information. Lower limb alignment during standing was evaluated using biplanar CR images obtained while participants stood with as extended as possible of the knees and toes in the neutral state. The OA knees showed flexion and varus both in the supine and standing positions, and neutral rotation of the tibia to the femur in the supine position and internal rotation of the tibia in the standing position. Fujii et al.’s study used compatible methods and experimental conditions with our study, and their results agreed well with those of our study. Both studies indicates that weight-bearing alters the 3D lower limb alignment of OA knees, particularly internal rotation of the tibia occurs in the standing position, although their study did not show the results with each OA grade.

To our knowledge, ours is the first study to demonstrate changes in 3D alignment in patients with knee OA of various grades under natural full weight-bearing conditions.

The classification of knee OA is an essential clinical issue, particularly in terms of the diagnosis of early knee OA [1]. As described above, differentiating early knee OA using 2D radiographs is difficult owing to a very small difference in joint space width between K-L grades 1 and 2 [8]. Although cone-beam CT imaging depicted the joint space widths of OA knees under weight-bearing conditions with a high level of repeatability [9–11], even 3D images obtained using this technique could not easily distinguish early changes associated with knee OA. In our analysis, we observed an average difference of 2.6° in the tibial internal rotation under weight-bearing conditions between K–L grades 1 and 2 (Fig. 3). Given the high level of accuracy of 3D–3D registration based on CT (0.2° about the X-axis and Z-axis [18]), we could clearly visualise early changes associated with knee OA. The weight-bearing conditions led to increased flexion, adduction, and tibial internal rotation as the OA grade increased. Moreover, a significant increase in tibial internal rotation occurred prior to the increases in flexion and adduction, suggesting that internal rotation under weight-bearing conditions is a key pathologic factor in the progression of knee OA.

Several studies have depicted the 3D kinematics of OA knees during weight-bearing activities. Matsuki et al. used fluoroscopy and 2D–3D registration to evaluate the knee kinematics associated with early knee OA (K–L grades 1 and 2) during pivoting and squatting activities [24]. In that study, reduced tibial internal rotation was observed during pivot activity in early OA knees relative to control knees. Another study similarly reported reduced tibial internal rotation during various weight-bearing activities in knees with advanced OA (K–L grades 3 and 4) relative to healthy knees [25]. Both studies demonstrated differences in the tibiofemoral rotation patterns between OA and healthy knees. Till date, only one study has suggested a link between tibial rotation and the onset of knee OA. Andriacchi et al. used a computer simulation to demonstrate the effect of tibial internal rotation during gait on the thinning of cartilage in the knee [26]. The authors found that a 5° increase in tibial internal rotation, which is usually seen in the anterior cruciate ligament deficient knee, was associated with rapid rate cartilage thinning in the medial compartment and speculated that this increase could initiate knee OA. We also thought that ACL function causes the change in rotation from the supine to the upright position. In the past, it has been reported that ACL function declines by 20% between people in their 20s and 80s, and aging may be one of the reasons for ACL dysfunction [27]. In another upright cone-beam CT, arch drop was found to cause internal rotation of the tibia in a group of women at high risk for knee OA [28]. This suggests that tibial internal rotation may be a risk factor for more severe knee OA. It was suggested that abnormal rotation of the tibia triggered by ACL dysfunction may be the trigger of the OA and may determine the indication for early prevention of progression of the OA and surgeries such as high tibial osteotomy or total knee arthroplasty. However, the relation between abnormal tibial kinematics and the onset and progression of knee OA remains unclear and further investigation is needed.

Several limitations of this study should be noted. First, even when we performed a power analysis prior to the study, to analyse statistical difference in each OA grade, the statistical power of our analysis was limited by the small number of OA patients included in our study. Even with the limited numbers of the patients, our results clearly demonstrate potential to diagnose early-stage knee OA using upright CT. Second, to evaluate accurate tibial internal rotation in standing, as shown in the present study and in the study by Fuji et al. [12], CT images with matching technique are required [12]. As anterior-posterior radiograph is still the gold standard to diagnose knee OA, there is a need to develop method to evaluate tibial internal/external rotation on radiographs. In the future, computer-aided method with deep learning algorithm has a potential to evaluate the tibial rotation and to diagnose early-stage knee OA on 2D radiograph [29]. Third, K-L grading was done on supine non-weight-bearing radiographs. However, assessment of joint space narrowing (K-L grade 3 and 4) is certainly impaired by grading on non-weight-bearing images and the fully extended position also is known to reduce reliability and validity of assessments of joint space narrowing. Forth, we evaluated knee kinematics under weight-bearing conditions while the subjects stood on both legs. Under this physiological condition, the entire body weight was divided evenly between the knees. However, the application of greater weight-bearing conditions would likely elucidate the pathology of knee OA. Moreover, using a self-selected pose could lead to patients with more severe knee OA standing in a way that minimized discomfort. Future studies should aim to examine knee kinematics under various weight-bearing conditions and at different knee flexion angles.

## Conclusion

Changes in the 3D alignment of the knee under weight-bearing conditions were assessed in patients with knee OA via a novel 320-row upright CT protocol. Changes in 3D deformities (flexion, adduction, and tibial internal rotation) from a non-weight-bearing to weight-bearing condition were observed in end-stage OA knees. No differences were found in flexion and adduction between K-L grade 1 and 2 knees, but greater tibial internal rotation was observed in K-L grade 2 knees as opposed to K-L grade 1 knees when weight-bearing. These results suggest that greater tibial rotation when weight-bearing is a key consideration to differentiate early-stage patients from advanced stage patients.

## Data Availability

The datasets of this study are available from the corresponding author upon reasonable request.

## Abbreviations

OA: Osteoarthritis
2D: Two-dimensional
K–L: Kellgren–Lawrence
CT: Computed tomography
3D: Three-dimensional
ICP: Iterative closest point
CR: Computed radiography

## References

[1] Madry H, Kon E, Condello V, Peretti GM, Steinwachs M, Seil R, Berruto M, Engebretsen L, Filardo G, Angele P (2016). Early osteoarthritis of the knee. Knee Surg Sports Traumatol Arthrosc.

[2] Kellgren JH, Lawrence JS (1957). Radiological assessment of osteo-arthrosis. Ann Rheumatic Dis.

[3] Damen J, Schiphof D, Wolde ST, Cats HA, Bierma-Zeinstra SM, Oei EH (2014). Inter-observer reliability for radiographic assessment of early osteoarthritis features: the CHECK (cohort hip and cohort knee) study. Osteoarthritis Cartilage.

[4] Scott WW, Lethbridge-Cejku M, Reichle R, Wigley FM, Tobin JD, Hochberg MC (1993). Reliability of grading scales for individual radiographic features of osteoarthritis of the knee. The Baltimore longitudinal study of aging atlas of knee osteoarthritis. Invest Radiol.

[5] Altman RD, Gold GE. Atlas of individual radiographic features in osteoarthritis, revised. Osteoarthritis Cartilage. 2007;15(Suppl A):A1–56. 10.1016/j.joca.2006.11.009 17320422

[6] Altman RD, Hochberg M, Murphy WA, Wolfe F, Lequesne M (1995). Atlas of individual radiographic features in osteoarthritis. Osteoarthritis Cartilage.

[7] Oka H, Muraki S, Akune T, Mabuchi A, Suzuki T, Yoshida H, Yamamoto S, Nakamura K, Yoshimura N, Kawaguchi H (2008). Fully automatic quantification of knee osteoarthritis severity on plain radiographs. Osteoarthritis Cartilage.

[8] Oka H, Muraki S, Akune T, Nakamura K, Kawaguchi H, Yoshimura N (2010). Normal and threshold values of radiographic parameters for knee osteoarthritis using a computer-assisted measuring system (KOACAD): the ROAD study. J Orthopaedic Sci.

[9] Segal NA, Bergin J, Kern A, Findlay C, Anderson DD (2017). Test-retest reliability of tibiofemoral joint space width measurements made using a low-dose standing CT scanner. Skeletal Radiol.

[10] Segal NA, Frick E, Duryea J, Nevitt MC, Niu J, Torner JC, Felson DT, Anderson DD (2017). Comparison of tibiofemoral joint space width measurements from standing CT and fixed flexion radiography. J Orthop Res.

[11] Hirschmann A, Buck FM, Fucentese SF, Pfirrmann CW (2015). Upright CT of the knee: the effect of weight-bearing on joint alignment. Eur Radiol.

[12] Fujii T, Sato T, Ariumi A, Omori G, Koga Y, Endo N. A comparative study of weight-bearing and non-weight-bearing 3-dimensional lower extremity alignment in knee osteoarthritis. J Orthop Sci. 2020;25(5):874–9. 10.1016/j.jos.2019.11.012 31955959

[13] Jinzaki M, Yamada Y, Nagura T, Nakahara T, Yokoyama Y, Narita K, et al. Development of Upright Computed Tomography With Area Detector for Whole-Body Scans: Phantom Study, Efficacy on Workflow, Effect of Gravity on Human Body, and Potential Clinical Impact. Invest Radiol. 2020;55(2):73–83. 10.1097/RLI.0000000000000603 PMC6948833 31503082

[14] Ota T, Nagura T, Yamada Y, Yamada M, Yokoyama Y, Ogihara N, Matsumoto M, Nakamura M, Jinzaki M (2019). Effect of natural full weight-bearing during standing on the rotation of the first metatarsal bone. Clin Anat.

[15] Kaneda K, Harato K, Oki S, Ota T, Yamada Y, Yamada M, Matsumoto M, Nakamura M, Nagura T, Jinzaki M (2019). Three-dimensional kinematic change of hindfoot during full weightbearing in standing: an analysis using upright computed tomography and 3D-3D surface registration. J Orthop Surg Res.

[16] Yamada Y, Yamada M, Yokoyama Y, Tanabe A, Matsuoka S, Niijima Y, et al. Differences in Lung and Lobe Volumes Between Supine and Standing Positions Scanned with Conventional and Newly Developed 320-Detector-Row Upright CT: Intra-Individual Comparison. Respiration. 2020;99(7):598–605. 10.1159/000507265 PMC7490509 32640453

[17] Besl PJ, McKay ND (1992). A method for registration of 3-D shapes. IEEE Trans Pattern Analysis Machine Intelli.

[18] Ochia RS, Inoue N, Renner SM, Lorenz EP, Lim TH, Andersson GB, An HS (2006). Three-dimensional in vivo measurement of lumbar spine segmental motion. Spine (Phila Pa 1976).

[19] Sato T, Koga Y, Sobue T, Omori G, Tanabe Y, Sakamoto M (2007). Quantitative 3-dimensional analysis of preoperative and postoperative joint lines in total knee arthroplasty: a new concept for evaluation of component alignment. J Arthroplasty.

[20] Enomoto H, Nakamura T, Waseda A, Niki Y, Toyama Y, Suda Y (2013). A novel and reproducible reference axis for distal tibial axial rotation. J Arthroplasty.

[21] Wu G, Siegler S, Allard P, Kirtley C, Leardini A, Rosenbaum D, Whittle M, D'Lima DD, Cristofolini L, Witte H (2002). ISB recommendation on definitions of joint coordinate system of various joints for the reporting of human joint motion--part I: ankle, hip, and spine. International Society of Biomechanics. J Biomechanics.

[22] Akagi M, Oh M, Nonaka T, Tsujimoto H, Asano T, Hamanishi C (2004). An anteroposterior axis of the tibia for total knee arthroplasty. Clin Orthop Relat Res.

[23] Matsui Y, Kadoya Y, Uehara K, Kobayashi A, Takaoka K (2005). Rotational deformity in varus osteoarthritis of the knee: analysis with computed tomography. Clin Orthop Related Res.

[24] Matsuki K, Matsuki KO, Kenmoku T, Yamaguchi S, Sasho T, Banks SA (2017). In vivo kinematics of early-stage osteoarthritic knees during pivot and squat activities. Gait Posture.

[25] Hamai S, Moro-oka TA, Miura H, Shimoto T, Higaki H, Fregly BJ, Iwamoto Y, Banks SA (2009). Knee kinematics in medial osteoarthritis during in vivo weight-bearing activities. J Orthop Res.

[26] Andriacchi TP, Briant PL, Bevill SL, Koo S (2006). Rotational changes at the knee after ACL injury cause cartilage thinning. Clin Orthop Related Res.

[27] Woo SL, Hollis JM, Adams DJ, Lyon RM, Takai S (1991). Tensile properties of the human femur-anterior cruciate ligament-tibia complex. The effects of specimen age and orientation. Am J Sports Med.

[28] Rabe KG, Segal NA, Waheed S, Anderson DD (2018). The Effect of Arch Drop on Tibial Rotation and Tibiofemoral Contact Stress in Postpartum Women. PM R.

[29] Almeida DF, Astudillo P, Vandermeulen D (2021). Three-dimensional image volumes from two-dimensional digitally reconstructed radiographs: A deep learning approach in lower limb CT scans. Med Phys.

